# Association of Ethnicity, Sex, and Age With Cancer Diagnoses and Health Care Utilization Among Children in Inner Mongolia, China

**DOI:** 10.1001/jamanetworkopen.2022.31182

**Published:** 2022-09-12

**Authors:** Hu-Zi-Wei Zhou, Li-Ying Qiao, Yun-Jing Zhang, Wei-Wei Kang, Xue Yan, Yu-Ling Jiang, Ya-Lei Ke, Ying-Ting Rao, Guo-Zhen Liu, Ming-Yuan Wang, Hui Wang, Yun-Feng Xi, Sheng-Feng Wang

**Affiliations:** 1 Department of Epidemiology and Biostatistics, School of Public Health, Peking University, Beijing, China; 2 Department of Chronic Noncommunicable Diseases Prevention and Control, The Inner Mongolia Autonomous Region Comprehensive Center for Disease Control and Prevention, Inner Mongolia, China; 3 School of Public Health, Baotou Medical College, Baotou, China; 4 Bigdata Division, Innovation Center, Peking University Health Information Technology, Beijing, China; 5 Department of Maternal and Child Health, School of Public Health, Peking University, Beijing China

## Abstract

**Importance:**

China is experiencing a sustained increase in childhood cancer. However, whether differences exist in disease burden by ethnicity remains unclear.

**Objective:**

To compare differences in cancer diagnoses and health care utilization in Inner Mongolia among children subgrouped by ethnicity (Han vs Mongolian), sex, and age.

**Design, Setting, and Participants:**

This retrospective cohort study in Inner Mongolia, China, used data on children aged 0 to 14 years with cancer from the Inner Mongolia Regional Health Information Platform, which comprises the National Basic Medical Insurance database and the Inner Mongolia cause-of-death reporting system, from January 1, 2013, to December 31, 2019. Ethnicities analyzed included Han and Mongolian; patients of other ethnicities were not included in the analysis because of the small sample size. Cancer was broadly defined as a primary malignant tumor or hematologic cancer; benign central nervous system tumors were also included. A 2-year washout period was used to exclude prevalent cases. After diagnosis, the patients were followed up until the date of death or the end of the insured status, whichever came first.

**Exposures:**

Ethnicity (Han vs Mongolian), sex (male vs female), and age (0-4, 5-9, and 10-14 years).

**Main Outcomes and Measures:**

Crude incidence, 5-year prevalence, and survival rates at 1 year and 3 years after diagnosis; health care utilization, represented by medical costs during the first year and first 3 years after diagnosis; and hospital attendance with level (tertiary vs secondary and lower-level hospitals) and location of each unique visit.

**Results:**

From 2013 to 2019, 1 106 684 (2013), 1 330 242 (2014), 1 763 746 (2015), 2 400 343 (2016), 2 245 963 (2017), 2 901 088 (2018), and 2 996 580 (2019) children aged 0 to 14 years were registered in the NBMI database. Among the 2 996 580 children enrolled in 2019, the mean (SD) age was 6.8 (4.3) years, of whom 1 572 096 (52.5%) were male, 2 572 091 (85.8%) were Han, and 369 400 (12.3%) were Mongolian. A total of 1910 patients with cancer were identified (1048 were male [54.9%]; 1559 were Han [81.6%], and 300 were Mongolian [15.7%]). There were 764 hematologic cancers (40.0%) and 1146 solid tumors (60.0%). The overall crude incidence of cancer from 2015 to 2019 was 129.85 per million children (95% CI, 123.63-136.06), with a higher incidence among Mongolian than among Han children (155.12 [95% CI, 136.81-173.43] vs 134.39 [95% CI, 127.46-141.32]). The 5-year prevalence was 428.97 per million (95% CI, 405.52-452.42) in 2020, with a higher prevalence among Mongolian than among Han children (568.49 [95% CI, 91.62-645.36] vs 404.34 [95% CI, 379.77-428.91]). The combined 1-year (2015-2019) and 3-year (2015-2017) survival rates were 72.5% (95% CI, 67.5%-77.5%) and 66.8% (95% CI, 61.6%-71.9%), respectively. The 1-year (median [IQR], $1991 [$912-$10 181] vs $3991 [$1171-$15 425]) and 3-year (median [IQR], $2704 [$954-$13 909] vs $5375 [$1283-$22 466]) postdiagnosis costs were lower among Mongolian than among Han children. A higher proportion of Mongolian patients attended low-level hospitals (45.9% vs 17.4%).

**Conclusions and Relevance:**

In this cohort study, Mongolian children had a higher incidence and prevalence of cancer but a lower demand for medical care, suggesting that further investigations are needed to identify mechanisms underlying ethnic disparities and ensure that care is equitable.

## Key Points

**Question:**

Do disparities exist in the burden of childhood cancer between Mongolian and Han children in Inner Mongolia, China?

**Findings:**

In this cohort study of 1910 patients aged 0 to 14 years with cancer, children of Mongolian ethnicity had a significantly higher incidence (155.12 vs 134.39 per million) and 5-year prevalence (568.49 vs 404.34 per million) but significantly lower medical costs during 1-year and 3-year postdiagnosis periods than Han children. A greater proportion of Mongolian children also attended lower-level hospitals.

**Meaning:**

These results suggest that more efforts should be made to understand the underlying causes of disparities in disease burden to achieve health equity.

## Teaser

This cohort study uses data from a national medical insurance database to assess the associations of ethnicity, sex, and age with childhood cancer in Inner Mongolia.

## Introduction

Childhood cancer, with an estimated 400 000 diagnoses worldwide per year,^1^ rising incidence,^1^ unequal access to treatment,^2,3^ high medical costs,^4^ enormous cost in disability-adjusted life-years,^5^ poor prognosis in the absence of cancer-directed therapy,^6^ and significant long-lasting influences,^6^ imposes a serious disease burden on children worldwide. Since the 1990s, there has been an increase in ethnicity-related childhood cancer studies, leading to increased attention on racial and ethnic heterogeneity.^7^ Previous studies^8,9,10,11,12^ found that ethnic minority groups tend to be more susceptible to severe disease progression among certain childhood cancers.

As the largest developing and most populous country in the world, China has the greatest number of childhood cancer cases globally, estimated to be more than 45 000 new cases per year.^2^ In fact, in 2019, cancer was the second leading cause of death among children aged 1 to 14 years.^13^ Furthermore, China is home to 120 million members of ethnic minority groups, a number that is increasing. The seventh national census, completed in 2021, showed that China’s ethnic minority population has increased by 10.26% from a decade ago, with a 0.4% increase in population share.^14^ However, limited research has focused on the ethnicity-specific childhood cancer burden in China.^9,15,16,17^ Unfortunately, previous population-based studies failed to consider the burden of health care utilization, which partly reflects health care accessibility and disease prognosis.

As the third largest province in China, Inner Mongolia has a population of 24 million, of whom 4.25 million are of Mongolian ethnicity, accounting for over 70% of the Mongolian population in China and 50% in the world.^18^ Inner Mongolia implemented a universal medical insurance system based on the National Basic Medical Insurance (NBMI) in 2009, covering over 95% of the population by 2020. Therefore, we aimed to evaluate ethnic, sex, and age differences in disease frequency and health care utilization associated with childhood cancer in Inner Mongolia.

## Methods

This cohort study was approved by the Medical Ethics Committee of the Inner Mongolia Center for Disease Control and Prevention, and the need for informed consent from participants was waived because data were deidentified for this study; engineers, who signed data confidentiality agreements, disabled data analysts’ ability to track specific individuals by irreversibly encrypting identification numbers and masking names. This study followed the Strengthening the Reporting of Observational Studies in Epidemiology (STROBE) reporting guideline for cohort studies.

### Data Sources

Data were obtained from the Inner Mongolia Regional Health Information Platform from January 1, 2013, to December 31, 2019. The population of interest was children aged 0 to 14 years. The Inner Mongolia Regional Health Information Platform comprises the NBMI and the cause-of-death reporting system (CDRS) in Inner Mongolia. The NBMI contains the Urban Employee Basic Medical Insurance database, which covers working and retired employees in cities, and the Urban Resident Basic Medical Insurance database, which covers unemployed urban and rural residents (including children, students, and older adults, among others). These details have been previously described.^19^ The NBMI provides demographic characteristics, disease diagnoses, and cost information of participants, while death status was obtained from the CDRS via a link to the patient identification number. Patients with unknown sex in the NBMI were excluded (missing rate, 0.002%-0.457% during January 1, 2013, to December 31, 2019). To quantify the underreporting of the CDRS system in each year, the Inner Mongolia Autonomous Region Comprehensive Center for Disease Control and Prevention conducted 2 underreporting surveys of the whole province from 2015 to 2017 and from 2018 to 2020, using the capture-recapture method.^20^ The proportion of garbage codes (for implausible or nonspecific causes of death) in the CDRS was at an acceptable level during 2014 to 2019 (eTable 1 in the Supplement). After diagnosis, the patients were followed up until the date of death or the end of the insured status, whichever came first.

### Categories of Cancers

Childhood cancers were defined broadly as hematologic cancers or solid tumors. Primary malignant tumors, hematologic cancers (leukemia and lymphoma), and benign tumors of the central nervous system were then categorized into 12 diagnostic groups according to the *International Classification of Childhood Cancers, Third Edition*.^21^ A manual review of the cancer diagnoses was performed (H.Z.W.Z. and Y.J.Z). Initially, all records containing the Chinese characters referring to cancer: *cancer*, *tumor*, *carcinoma*, *sarcoma*, *malignancy*, *leukemia*, *lymphoma*, and other possible cancer diagnoses from patients’ medical records were extracted.^22^ On this basis, nonindex cancers (including nonmalignant or nonmetastatic tumors, precancerous lesions, and other nonneoplastic diseases) were excluded with the assistance of pediatricians (including H.W.). Tumors without specific sites were classified as other and unspecified malignant tumors. With a 2-year washout period to exclude prevalent cases, an incident case was defined as the first definite index-cancer record, with the corresponding admission date as the onset date. Consistent with the methods described by Tian et al,^22^ only the first definitive cancer diagnosis during the study period was retained as a cancer type classification for each patient.

### Statistical Analysis

Disease frequency was described as incidence (incidence rate per 1 million children), 5-year prevalence (per 1 million children), and survival rate (at 1 year, 3 years, and 5 years) using the following formulas: incidence, (number of new cases of cancer during a given period ÷ population at risk during a given period) × 1 million; 5-year prevalence rate, (number of children alive who had a cancer diagnosis during the past 5 years ÷ pediatric population aged 0-14 years) × 1 million; and survival rates, (number of patients alive more than N years after diagnosis ÷ number of patients diagnosed that year) × 100.

Sociodemographic variables included sex (male or female), age (0-4; 5-9; and 10-14 years), and ethnic group (Han or Mongolian). As Han and Mongolian individuals made up 96.81% of the children in Inner Mongolia, members of all other ethnic minority groups were categorized as other and were excluded from further analysis for the sake of relatively reliable rates estimation.^23,24^ Ethnic classification was confirmed by the participant or participant’s parent or guardian when registering for the NBMI. Age-standardized rates were determined using the World Health Organization world standardized rates (WSRs), 2000 to 2025.^25^

Health care utilization was analyzed by medical costs related to cancer during the first year and the first 3 years after diagnosis and hospital attendance of incident cases. Costs were discounted through the Consumer Price Index to remove the impact of inflation and were shown at the 2019 level (US $1.00 = 6.87 RMB on July 1, 2019). Hospital attendance was characterized by the number of hospitals that patients attended throughout their visits, as well as the levels of care and hospital location; according to Chinese hospital management standards, hospitals are classified by level, as tertiary hospitals, secondary and lower-level, and by location, as inside or outside Inner Mongolia.

To explore associations with income, living conditions, and lifestyles, subgroup analyses were conducted by city, 2-level strata (high or low) according to gross domestic product (GDP) per capita (GDP divided by total population), grassland area share, alcohol consumption rate, and smoking rate. Data on alcohol consumption and smoking rates were obtained from the 2018 Adult Chronic Disease and Nutrition Monitoring Survey in Inner Mongolia. A sensitivity analysis was performed to assess the effect of death underreporting by recalculating the number of patients diagnosed who died in 2015 to 2019 based on the underreporting rate from the 2 CDRS completeness surveys.^20^ The χ^2^ test, Wilcoxon rank test, and Kruskal-Wallis test were used to compare differences in the characteristics of interest. Two-sided *P* < .05 was considered statistically significant. All statistical analyses were performed using R statistics software, version 4.0.1 (R Foundation for Statistical Computing).

## Results

### Demographic Characteristics

From 2013 to 2019, 1 106 684 (2013), 1 330 242 (2014), 1 763 746 (2015), 2 400 343 (2016), 2 245 963 (2017), 2 901 088 (2018), and 2 996 580 (2019) children aged 0 to 14 years were registered in the NBMI database. Among the 2 996 580 children enrolled in 2019, the mean (SD) age was 6.8 (4.3) years, of whom 1 572 096 (52.5%) were male, 2 572 091 (85.8%) were Han, of whom 1 455 135 (56.6%) lived in cities with low GDP per capita; 369 400 (12.3%) were Mongolian, of whom 324 505 (87.8%) lived in cities with low GDP per capita. Overall, 1910 patients with cancer were identified (1048 male [54.9%] and 862 female [45.1%]; ratio, 1.22:1); 1559 patients (81.6%) were Han and 300 (15.7%) were Mongolian. There were 764 (40.0%) hematologic cancers and 1146 (60.0%) solid tumors. The most common cancer types were leukemia (n = 637 [33.4%]), central nervous system tumors (209 [10.9%]), and lymphoma (127 [6.6%]) (Table 1). Sixty-one patients (3.2%) with discontinued NBMI payment status were unavailable for follow-up.

**Table 1.** Characteristics of Patients With Childhood Cancer in Inner Mongolia, 2013 to 2019

Characteristic	Participants, No. (%)			*P* value
	Overall^a^	Han	Mongolian	
Total	1910	1559 (81.6)	300 (15.7)	
Age at first time visit, y				
Mean (SD)	6.3 (4.1)	6.3 (4.0)	5.8 (4.3)	.05
Median (IQR)	6.0 (3.0-10.0)	6.1 (3.0-10.1)	5.0 (2.0-9.0)	
Age group				
0-4	793 (41.5)	634 (40.7)	136 (45.3)	<.001
5-9	602 (31.5)	494 (31.7)	92 (30.7)	
10-14	515 (27.0)	431 (27.6)	72 (24.0)	
Sex				
Male	1048 (54.9)	839 (53.8)	183 (61.0)	.03
Female	862 (45.1)	720 (46.2)	117 (39.0)	
Cancer type				
Hematologic cancer	764 (40.0)	631 (40.5)	114 (38.0)	.46
Malignant tumor	1146 (60.0)	928 (59.5)	186 (62.0)	
Main cancer diagnosis				
Leukemia	637 (33.4)	527 (33.8)	95 (31.7)	.79
Lymphoma	127 (6.6)	104 (6.7)	19 (6.3)	
CNS tumor	209 (10.9)	173 (11.1)	31 (10.3)	
Other solid tumor	901 (47.2)	755 (48.4)	155 (51.7)	
Hospital, by first time visit				
Secondary and lower-level hospitals	490 (25.6)	342 (22.0)	135 (45.0)	<.001
Tertiary hospitals	1420 (74.4)	1217 (78.0)	165 (55.0)	
Inner Mongolia				
Outside	997 (52.2)	779 (50.0)	90 (30.0)	<.001
Inside	913 (47.8)	780 (50.0)	210 (70.0)	

Abbreviation: CNS, central nervous system.

^a^ Because of the small sample size, the 41 patients (2.1%) from minority groups other than Han or Mongolian were not included in the ethnicity-specific analysis so a relatively reliable estimation of the rate could be obtained.

### Incidence, 2015 to 2019

From January 1, 2015, to December 31, 2019, 1676 new cancer diagnoses occurred, with a crude incidence of 129.85 (95% CI, 123.63-136.06) and a WSR of 136.77 (95% CI, 130.39-143.15) per million children (Table 2). A higher incidence of cancer diagnosis was found among males than in females (929 cancers; crude incidence, 144.16 [95% CI, 135.10-153.22] vs 747 cancers; crude incidence 121.35 [95% CI, 112.65-130.06] per million children), among children aged 0 to 4 years (725 cancers; crude incidence, 206.08 [95% CI, 191.08-221.08] per million children) than in those 5 to 9 years (519 cancers; crude incidence, 109.46 [95% CI, 100.05-118.88] per million) and 10 to 14 years (432 cancers; crude incidence, 92.94 [95% CI, 84.18-101.70] per million), and among Mongolian children than Han children (270 cancers; crude incidence, 155.12 [95% CI, 136.81-173.43] per million vs 1365 cancers; crude incidence, 134.39 [95% CI, 127.46-141.32] per million). In subgroup analyses, ethnic disparity remained across most stratifications but was not stable at the city level (eTables 2 and 3 in the Supplement).

**Table 2.** Incidence of Childhood Cancers During 2015-2019 in Inner Mongolia, per Million Children

	Overall incidence^a^			Males			Females		
	New cancer diagnoses, No.	Crude rate (95% CI)	WSR (95% CI)^b^	New cancer diagnoses, No.	Crude rate (95% CI)	WSR (95% CI)	New cancer diagnoses, No.	Crude rate (95% CI)	WSR (95% CI)
Overall	1676	129.85 (123.63-136.06)	136.77 (130.39-143.15)	929	137.59 (128.74-146.44)	144.16 (135.1-153.22)	747	121.35 (112.65-130.06)	128.62 (119.66-137.58)
Age group, y									
0-4	725	206.08 (191.08-221.08)	NA	397	213.43 (192.44-234.42)	NA	328	197.83 (176.42-219.23)	NA
5-9	519	109.46 (100.05-118.88)	NA	300	121.25 (107.53-134.97)	NA	219	96.60 (83.81-109.39)	NA
10-14	432	92.94 (84.18-101.70)	NA	232	95.96 (83.61-108.31)	NA	200	89.67 (77.24-102.09)	NA
Ethnic group									
Han	1365	127.11 (120.37-133.86)	134.39 (127.46-141.32)	741	131.65 (122.17-141.12)	137.97 (128.27-147.67)	624	122.12 (112.54-131.70)	130.53 (120.62-140.44)
Mongolian	270	152 (133.87-170.13)	155.12 (136.81-173.43)	164	178.09 (150.83-205.34)	182.07 (154.51-209.63)	106	123.91 (100.32-147.50)	126.02 (102.23-149.81)

Abbreviations: NA, not applicable; WSR, world standardized rate.

^a^ Because of the small sample size, the 31 patients (1.6%) from minority groups other than Han or Mongolian were not included in the ethnicity-specific analysis so a relatively reliable estimation of the rate could be obtained.

^b^ WSR is the age-standardized rate by world standard population (per 100 000).

### Five-Year Prevalence, 2018 to 2020

**Figure 1.**
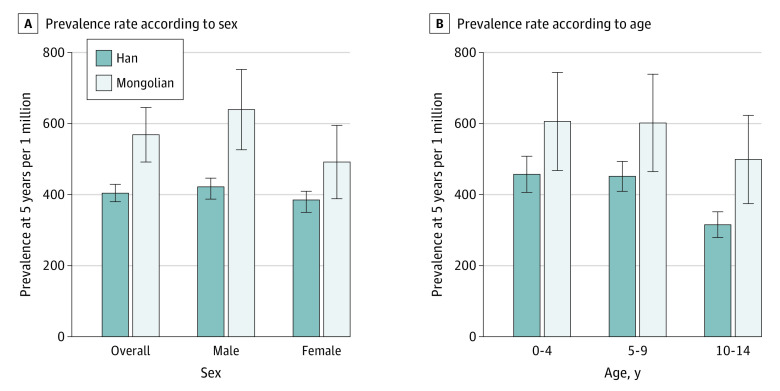
Crude 5-Year Childhood Cancer Prevalence Rates Among Han and Mongolian Patients With Childhood Cancer in 2020 Error lines are 95% CIs.

Age-standardized 5-year prevalence rates per 1 million children (determined using WSRs) from 2018 to 2020 were 263.75 (95% CI, 244.87-282.63) in 2018, 377.18 (95% CI, 354.85-399.51) in 2019, and 428.97 (95% CI, 405.52-452.42) in 2020 (eTable 4 and eTable 5 in the Supplement). The prevalence rates per 1 million children were higher in males (289.17 [95% CI, 261.88-316.46] in 2018, 405.13 [95% CI, 373.19-437.07] in 2019, and 454.21 [95% CI, 420.9-487.52]) than in females (235.76 [95% CI, 209.88-261.64] in 2018, 346.46 [95% CI, 315.40-377.52] in 2019, and 401.26 [95% CI, 368.37-434.15] in 2020). In 2020, there were significant disparities in prevalence between Han and Mongolian patients (crude rate, 404.34 [95% CI, 379.77-428.91] vs 568.49 [95% CI, 491.62-645.36] per 1 million children), which persisted across sex and age groups (Figure 1). Leukemia remained the most prevalent cancer type among all age and sex subgroups (eTable 6 in the Supplement). Interethnic prevalence rates were similar to the incidence rates between subgroups (eFigure 1 and eTable 7 in the Supplement). According to the 2 CDRS completeness surveys,^20^ the underreporting rates of the CDRS in 2015 to 2017 and 2018 to 2020 were 7.9% and 5.7%, respectively. The sensitivity analysis also suggested similar results, with only a slight decrease (eTable 5 in the Supplement).

### Survival Rates, 2015 to 2019

The overall 5-year survival rate from 2015 to 2019 was 70.6%, with the combined 1-year survival rate for 2015 to 2019 being 72.5% (95% CI, 67.5%-77.5%) and the combined 3-year survival rate for 2015 to 2017 being 66.8% (95% CI, 61.6%-71.9%). The temporal trends were relatively stable (eTable 8 in the Supplement); no distinct differences were observed between ethnic groups, and the sensitivity analysis suggested similar results.

### Health Care Utilization

The median (IQR) medical costs were $3621 ($1086-$14 535) during the first year after diagnosis and $5009 ($1207–$21 748) during the first 3 years (Table 3). The 1-year and 3-year postdiagnosis costs were relatively similar for patients across sex and age but were lower for Mongolian patients than for Han patients (median [IQR], $1991 [$912-$10 181] vs $3991 [$1171-$15 425] during the first year and median [IQR], $2704 [$954-$13 909] vs $5375 [$1283-$22 466] during first 3 years). Similar differences between the 2 ethnic groups were observed in most subgroups (ie, by city, GDP per capita, grassland area share, alcohol consumption rate, and smoking rate) (eTables 9 and 10 in the Supplement).

**Table 3.** Medical Costs for Patients With Childhood Cancer During the First Year and First 3 Years After Diagnosis in Inner Mongolia, 2015 to 2019

	Cost, median (IQR), $^a^			
	1 y After diagnosis	P value	3 y After diagnosis	P value
Overall	3621 (1086-14 535)	NA	5009 (1207-21 748)	NA
Sex				
Male	3835 (1037-15 310)	.75	5231 (1136-21 822)	.68
Female	3400 (1184-14 073)		4664 (1257-21 427)	
Age group, y				
0-4	3760 (1116-13 756)	.88	5017 (1239-19 584)	.20
5-9	3728 (1097-15 675)		5302 (1365-23 157)	
10-14	3175 (1074-15 385)		3831 (1081-20 764)	
Ethnic group^b^				
Han	3991 (1171-15 425)	<.001	5375 (1283-22 466)	<.001
Mongolian	1991 (912-10 181)		2704 (954-13 909)	

Abbreviation: NA, not applicable.

^a^ Costs are discounted based on the Consumer Price Index; the renminbi-to-US dollar exchange rate is based on the July 1, 2019, exchange rate (US $1.00 = 6.87 RMB).

^b^ Because of the small sample size, the 31 patients (1.6%) from minority groups other than Han or Mongolian were not included in the ethnicity-specific analysis so a relatively reliable estimation of the rate could be obtained.

**Figure 2.**
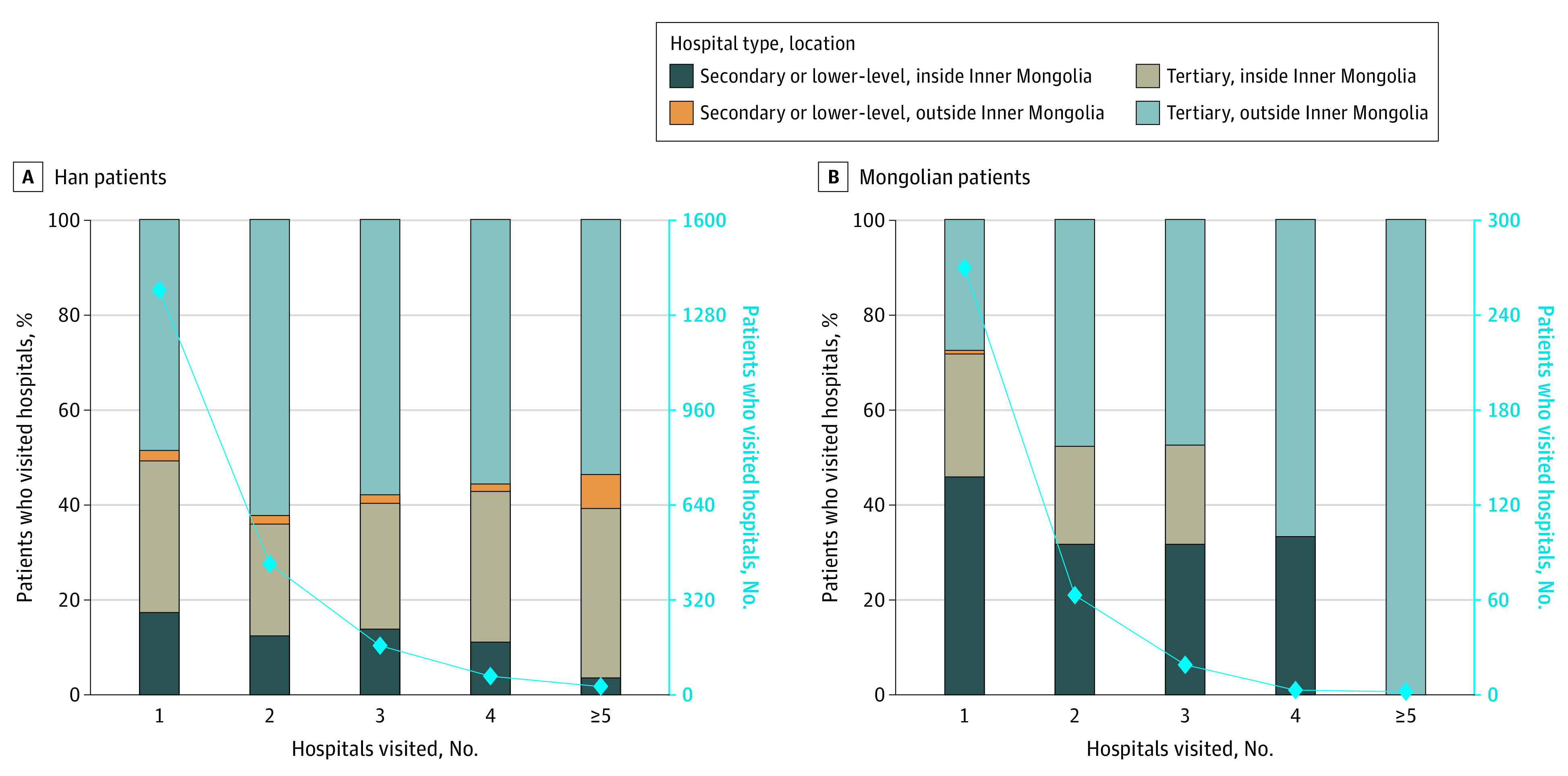
Hospital Flow of Han and Mongolian Patients With Childhood Cancer During Cancer Treatment Course The solid blue line shows the number of patients who visited the corresponding number of hospitals during the entire course of cancer treatment.

Throughout the course of cancer treatment, 516 patients (30.8%) had changed hospitals at least once (Figure 2). During each hospital visit throughout the cancer treatment, the percentage of Mongolian patients visiting secondary and lower-level hospitals was up to 45.9%, while this percentage was only up to 17.4% among Han patients. After stratification by GDP per capita, a similar phenomenon was observed in cities with low GDP per capita, which included most Mongolian patients (eFigure 2 in the Supplement).

## Discussion

This cohort study is, to the best of our knowledge, the first study in China to consider ethnic factors in the disease frequency and health care utilization of childhood cancer. Based on a large-scale integrated medical information database, we found that Mongolian children had higher incidence and 5-year prevalence than Han children but lower medical costs during 1- and 3-year postdiagnosis periods, as well as a greater likelihood of attending lower-level hospitals.

Our study found an overall incidence of 136.77 per million of childhood cancers in Inner Mongolia during 2015 to 2019, which is similar to previous findings (131.9 per million children) from the International Incidence of Childhood Cancer during 1990 to 2013.^26^ The rates in our study were higher than those previously reported in developing countries (eg, 8.6 per 100 000 children in India and 5.6 per 100 000 children in Mongolia) but lower than those in Western countries (eg, 18.1 per per 100 000 children in Italy, 17.2 per 100 000 children in Canada and the US, and 17.6 per 100 000 children in Germany),^27^ which was in concert with the relative rank in previous Chinese studies.^28,29^ However, we also observed higher rates than those of recent studies in China, which had an estimated national incidence of 93.32 per million in 2011 to 2015, including 116.3 per million in Shanghai and 113.34 per million in Beijing.^29,30,31^ Similarly, our observed incidence and 5-year prevalence were also higher than those reported by the Global Cancer Observatory in 2020 for China (10.9 per 100 000 children; and 428.97 per million vs 36.1 per 100 000 children, respectively).^27^ A likely explanation is the differences in data sources, as all previous studies were based on cancer registry data. Given the widely recognized shortcomings of cancer registry data, the nonnegligible underreporting rate (up to 20% in some hospitals in Shanghai with the best registry quality in China) may contribute to a considerable portion of the variations.^32^ Moreover, information on migrant children, who account for up to 12.8% of the children in China, is typically unavailable in cancer registries.^33^ Additionally, the incidence of childhood cancer is significantly increasing annually (up to 5.3% per year in China).^30^ Therefore, the observations in our study could be partially explained by a closer approximation to this trend. Nevertheless, possible associations of rates with regional differences cannot be ruled out. Previous studies in Inner Mongolia^34,35^ have indicated a higher prevalence of birth defects, which share pathophysiological features with childhood cancer, with approximately 9.2% of childhood cancers believed to be attributed to embryonic disorders. Therefore, more evidence regarding pathophysiological and environmental exposure conditions is required to interpret our findings.

Regarding the demographic distribution, males and children aged 0 to 4 years had a higher incidence of cancer, which is consistent with previous studies.^1,5,28^ This is, to our knowledge, the first study to report a higher incidence and prevalence of cancer in Mongolian children than in Han children, and the disparity remained after adjusting for region-level income, living conditions, and lifestyles (eTables 3 an 7 in the Supplement). Previous studies^36,37^ have reported that the incidences of liver cancer and hypertension were higher in the Mongolian population than in the Han population, but the association between pediatric diseases and ethnicity remains unknown. There may be several reasons for our findings. First, more alcohol consumption in daily life (regardless of sex) among Mongolian individuals may be a factor.^38^ In fact, alcohol consumption during pregnancy has been reported to be associated with an increased risk of leukemia or central nervous system tumors in offspring,^39^ which is also consistent with the cancer incidence disparity we observed between high and low levels of alcohol consumption across city groups (eTable 3 in the Supplement). Second, allergic diseases, such as food allergies and allergic rhinitis, which are more prevalent among Mongolian individuals, especially in pastoral areas, may also play a role.^40,41^ Although the overall association between childhood cancer and allergies remains unclear, a recent meta-analysis^42^ found that allergies may be associated with an increased incidence of acute lymphocytic leukemia in younger children. Third, the contribution of genetic differences cannot be excluded. For example, susceptibility polymorphisms associated with acute lymphocytic leukemia have been reported in European American, African American, and Hispanic populations, and acute lymphocytic leukemia–related differences in the frequency of chromosomal abnormalities and polymorphisms in folate metabolism pathways have been identified in several Asian populations.^43,44,45,46^ Recently, genomic variations among those with Mongolian and other Asian ethnic ancestry have been reported; however, the underlying genetic mechanisms associated with childhood cancer remain unclear.^47^

In our study, the medical costs during the 1-year postdiagnosis period for children with cancer in Inner Mongolia were $3621, much lower than those reported in Egyptian^48^ and Canadian^49^ studies during the same period ($14 774 and $137 693, respectively), but much closer to those in Shanghai ($2589 for solid tumors and $4229 for nonsolid tumors).^50^ Intercountry variation may be largely associated with differences in health care systems. Free treatment options for childhood cancer are available both in Canada and Egypt, whereas because of the deductible and reimbursement cap line of China’s NBMI system, patients undergoing long-cycle targeted therapy and some surgical options still incur relatively high medical costs even if granted partial reimbursement.^51^ Consequently, treatment abandonment may also contribute to comparatively low levels of medical costs among Chinese patients. In fact, in 2013, 62.6% of Chinese children with leukemia abandoned hematopoietic stem cell transplantation because of financial burden, with treatment abandonment rates reaching 10.0% for childhood cancer in 2015.^52,53^ Notably, Mongolian patients had lower costs than Han patients, which may be because Mongolian medicines (eg, such as combined chemotherapy drugs available in Mongolia for childhood cancer), prevailing in practice in this region, provide an additional option. However, most Mongolian medicine treatments were not covered by the NBMI until 2019, resulting in an underestimation of Mongolian patients’ costs.^54^

Another notable finding was that a higher proportion of Mongolian children attended lower-level hospitals. Multiple factors may be associated with this finding. For one, more Mongolian populations live in agricultural and pastoral areas, farther away from tertiary hospitals in urban centers.^55,56^ Increased distance may limit patients’ access to large urban medical centers, which is associated with an increased probability of traveling to closer hospitals.^57^ Second, Mongolian families still have a strong identification with and reliance on Mongolian medicine, and most Mongolian hospitals are not classified as tertiary.^54,56^ Moreover, an association with low household income cannot be excluded, as more Mongolian children lived in cities with a lower GDP per capita than Han children (87.8% compared with 56.6% for Han in 2019). However, the GDP per capita stratified results in low-GDP cities were similar, indicating that ethnic disparities in hospital flow cannot be ignored.

### Limitations

This study has several limitations. First, although our study has covered more than 90% of children in Inner Mongolia, because of the limited number of patients, especially Mongolian children, this study remains insufficient to support intergroup comparisons of disease burden by cancer type, and the small sample sizes for some strata made the subgroup analyses less stable. Nevertheless, our study suggests that interethnic differences in the disease burden of childhood cancer persist after stratification. Second, cause of death is inevitably underreported, leading to overestimation of prevalence and survival rates. However, data quality evaluations of the completeness and accuracy of the CDRS,^20^ as well as sensitivity analyses, would suggest that their impact on our data was minor. Third, although we did not validate cancer diagnoses in NBMI diagnostic records with laboratory tests because of privacy protection and data accessibility, previous studies using NBMI to identify cancer cases have demonstrated sensitivities and positive predictive values above 90%.^58,59^ Fourth, we were unable to capture socioeconomic and lifestyle-related variables at the individual level; thus the possibility of associations with these factors cannot be completely excluded. Fifth, our study was restricted to Inner Mongolia, and extrapolation of the findings needs to be confirmed by larger studies with longer follow-up.

## Conclusions

This large-scale population-based retrospective cohort study of the disease burden of childhood cancer and its demographic distribution in Inner Mongolia found an association between ethnic groups and disparities in childhood cancer incidence and prevalence. The incidence and prevalence in Mongolian patients were higher than those in Han patients, but the demand for medical care was lower in Han patients. These findings suggest that further studies are needed to examine the mechanisms of the association of ethnic factors with health disparities among children with cancer and to take practical steps to reduce health inequities.

## Notes

Supplement. **eTable 1.** Proportion of Garbage Codes in the Cause-of-Death-Reporting System in Inner Mongolia From 2014 to 2019 **eTable 2.** Incidence Rates of Childhood Cancer in Inner Mongolia During 2015-2019, Grouped by City and Ethnic Group, per Million Children **eTable 3.** Incidence Rates of Childhood Cancer in Inner Mongolia During 2015-2019, Grouped by GDP Per Capita Level/Grassland Area Share Level/Alcohol Consumption Rate Level/Smoking Rate Level, and Ethnic Group, per Million Children **eTable 4.** Five-Year Prevalence Rates of Childhood Cancer of 2018-2020 in Inner Mongolia, Grouped by Age, per Million Children **eTable 5.** Five-Year Prevalence Rates of Childhood Cancer of 2018-2020 in Inner Mongolia, Grouped by Sex and Ethnic Group, per Million Children **eTable 6.** Five-Year Prevalence Rates of Childhood Cancer of 2020 in Inner Mongolia, Grouped by Cancer Type, Sex, and Age, per Million Children **eFigure 1.** Five-Year Prevalence Rates of Childhood Cancer of 2018-2020 in Inner Mongolia, Grouped by City and Ethnic Group, per Million Children **eTable 7.** Five-Year Prevalence Rates of Childhood Cancer of 2018-2020 in Inner Mongolia, Grouped by GDP Per Capita Level/Grassland Area Share Level/Alcohol Consumption Rate Level/Smoking Rate Level, and Ethnic Group, per Million Children **eTable 8.** Survival Rates of Childhood Cancer of 2015-2019 in Inner Mongolia, Grouped by Year and Ethnic Group **eTable 9.** Medical Costs for Childhood Cancer Patients During the First Year and First 3 Years After Diagnosis in Inner Mongolia, 2015-2019, Grouped by City and Ethnic Group **eTable 10.** Medical Costs for Childhood Cancer Patients During the First Year and First 3 Years After Diagnosis in Inner Mongolia, 2015-2019, Grouped by GDP Per Capita Level/Grassland Area Share Level/Alcohol Consumption Rate Level/Smoking Rate Level, and Ethnic Group **eFigure 2.** Hospital Flow of Patients With Childhood Cancer During Cancer Treatment Course in Inner Mongolia, 2015-2019, Grouped by GDP Per Capita Level and Ethnic Group

## References

[1] Steliarova-Foucher E, Colombet M, Ries LAG, ; IICC-3 Contributors. International incidence of childhood cancer, 2001-10: a population-based registry study. Lancet Oncol. 2017;18(6):719-731. doi:10.1016/S1470-2045(17)30186-9 28410997 PMC5461370

[2] Rodriguez-Galindo C, Friedrich P, Alcasabas P, . Toward the cure of all children with cancer through collaborative efforts: pediatric oncology as a global challenge. J Clin Oncol. 2015;33(27):3065-3073. doi:10.1200/JCO.2014.60.6376 26304881 PMC4979198

[3] Pritchard-Jones K, Pieters R, Reaman GH, . Sustaining innovation and improvement in the treatment of childhood cancer: lessons from high-income countries. Lancet Oncol. 2013;14(3):e95-e103. doi:10.1016/S1470-2045(13)70010-X 23434338

[4] Lam CG, Howard SC, Bouffet E, Pritchard-Jones K. Science and health for all children with cancer. Science. 2019;363(6432):1182-1186. doi:10.1126/science.aaw4892 30872518

[5] Collaborators GCC; GBD 2017 Childhood Cancer Collaborators. The global burden of childhood and adolescent cancer in 2017: an analysis of the Global Burden of Disease Study 2017. Lancet Oncol. 2019;20(9):1211-1225. doi:10.1016/S1470-2045(19)30339-0 31371206 PMC6722045

[6] Bhakta N, Liu Q, Ness KK, . The cumulative burden of surviving childhood cancer: an initial report from the St Jude Lifetime Cohort Study (SJLIFE). Lancet. 2017;390(10112):2569-2582. doi:10.1016/S0140-6736(17)31610-0 28890157 PMC5798235

[7] Stiller CA, Parkin DM. Geographic and ethnic variations in the incidence of childhood cancer. Br Med Bull. 1996;52(4):682-703. doi:10.1093/oxfordjournals.bmb.a011577 9039726

[8] Delavar A, Barnes JM, Wang X, Johnson KJ. Associations between race/ethnicity and US childhood and adolescent cancer survival by treatment amenability. JAMA Pediatr. 2020;174(5):428-436. doi:10.1001/jamapediatrics.2019.6074 32091555 PMC7042928

[9] Friedrich P, Itriago E, Rodriguez-Galindo C, Ribeiro K. Racial and ethnic disparities in the incidence of pediatric extracranial embryonal tumors. J Natl Cancer Inst. 2017;109(10). doi:10.1093/jnci/djx050 29117360

[10] Mitchell HK, Morris M, Ellis L, Abrahão R, Bonaventure A. Racial/ethnic and socioeconomic survival disparities for children and adolescents with central nervous system tumours in the United States, 2000-2015. *Cancer Epidemiol*. 2020;64:101644. doi:10.1016/j.canep.2019.101644 31783249

[11] Haizel-Cobbina J, Spector LG, Moertel C, Parsons HM. Racial and ethnic disparities in survival of children with brain and central nervous tumors in the United States. *Pediatr Blood Cancer*. 2021;68(1):e28738. doi:10.1002/pbc.28738 32970937

[12] Zhao J, Han X, Zheng Z, et al. Racial/ethnic disparities in childhood cancer survival in the United States. *Cancer Epidemiol Biomarkers Prev*. 2021;30(11):2010-2017. doi:10.1158/1055-9965.EPI-21-0117 34593561

[13] Chinese Center for Disease Control and Prevention. Surveillance of Causes of Death Dataset of China, 2019. China Science and Technology Press; 2020.

[14] National Bureau Of Statistics. Seventh National Census Bulletin (No. 2). 2021MS08039. May 11, 2021. Accessed July 26, 2022. http://www.stats.gov.cn/english/PressRelease/202105/t20210510_1817185.html

[15] Whittle SB, Lopez MA, Russell HV. Payer and race/ethnicity influence length and cost of childhood cancer hospitalizations. Pediatr Blood Cancer. 2019;66(7):e27739. doi:10.1002/pbc.27739 30989762 PMC7057732

[16] Truong B, Green AL, Friedrich P, Ribeiro KB, Rodriguez-Galindo C. Ethnic, racial, and socioeconomic disparities in retinoblastoma. JAMA Pediatr. 2015;169(12):1096-1104. doi:10.1001/jamapediatrics.2015.2360 26436436

[17] Chow EJ, Puumala SE, Mueller BA, . Childhood cancer in relation to parental race and ethnicity: a 5-state pooled analysis. Cancer. 2010;116(12):3045-3053. doi:10.1002/cncr.25099 20564410 PMC2903004

[18] Inner Mongolia Bureau of Statistics. Inner Mongolia Autonomous Region Seventh National Population Census Bulletin (No. 1). May 20, 2021. Accessed February 20, 2022. https://www.nmg.gov.cn/tjsj/sjjdfx/202105/t20210526_1596820.html

[19] Zhang C, Feng J, Wang S, . Incidence of and trends in hip fracture among adults in urban China: a nationwide retrospective cohort study. PLoS Med. 2020;17(8):e1003180. doi:10.1371/journal.pmed.1003180 32760065 PMC7410202

[20] Stephen C. Capture-recapture methods in epidemiological studies. *Infect Control Hosp Epidemiol*. 1996;17(4):262-266. doi:10.1086/647290 8935735

[21] Steliarova-Foucher E, Stiller C, Lacour B, Kaatsch P. *International Classification of Childhood Cancer, Third Edition*. *Cancer*. 2005;103(7):1457-1467. doi:10.1002/cncr.20910 15712273

[22] Tian H, Yang W, Hu Y, . Estimating cancer incidence based on claims data from medical insurance systems in two areas lacking cancer registries in China. EClinicalMedicine. 2020;20:100312. doi:10.1016/j.eclinm.2020.100312 32215367 PMC7090368

[23] Emura T, Lin Y-S. A comparison of normal approximation rules for attribute control charts. Qual Reliab Eng Int. 2015;31:411-418. doi:10.1002/qre.1601

[24] Emura T, Liao YT. Critical review and comparison of continuity correction methods: the normal approximation to the binomial distribution. Commun Stat Simul Comput. 2018;47(8):2266-2285. doi:10.1080/03610918.2017.1341527

[25] Ahmad OB, Boschi Pinto C, Lopez A, Murray C, Lozano R, Inoue M. Age standardization of rates: a new WHO standard. EIP/GPE/EBD, World Health Organization. GPE Discussion Paper Series No. 31. 2001. Accessed September 30, 2021. https://cdn.who.int/media/docs/default-source/gho-documents/global-health-estimates/gpe_discussion_paper_series_paper31_2001_age_standardization_rates.pdf

[26] Steliarova-Foucher E, Colombet M, Ries LAG, Hesseling P, Moreno F, Shin HY, Stiller CA, eds. *International Incidence of Childhood Cancer*. Vol 3. International Agency for Research on Cancer; Updated April 11, 2017. Accessed July 25, 2021. https://iicc.iarc.fr/results

[27] International Agency for Research on Cancer. Global Cancer Observatory: cancer today: data visualization tools for exploring the global cancer burden in 2020. World Health Organization. Updated December 2020. Accessed August 9, 2022. https://gco.iarc.fr/today/online-analysis-table?v=2020&mode=population&mode_population=countries&population=900&populations=900&key=asr&sex=0&cancer=39&type=0&statistic=5&prevalence=0&population_group=0&ages_group%5B%5D=0&ages_group%5B%5D=2&group_cancer=1&include_nmsc=0&include_nmsc_other=1

[28] Zheng R, Peng X, Zeng H, . Incidence, mortality and survival of childhood cancer in China during 2000-2010 period: a population-based study. Cancer Lett. 2015;363(2):176-180. doi:10.1016/j.canlet.2015.04.021 25917566

[29] Sun K, Zheng R, Zhang S, . Patterns and trends of cancer incidence in children and adolescents in China, 2011-2015: a population-based cancer registry study. Cancer Med. 2021;10(13):4575-4586. doi:10.1002/cam4.4014 34076339 PMC8267116

[30] Yang L, Yuan Y, Sun T, Li H, Wang N. Characteristics and trends in incidence of childhood cancer in Beijing, China, 2000-2009. Chin J Cancer Res. 2014;26(3):285-292. 25035655 10.3978/j.issn.1000-9604.2014.06.09 PMC4076710

[31] Bao P-P, Zheng Y, Wang C-F, Gu K, Jin F, Lu W. Time trends and characteristics of childhood cancer among children age 0-14 in Shanghai. Pediatr Blood Cancer. 2009;53(1):13-16. doi:10.1002/pbc.21939 19260104

[32] Su C, Tang Z. Analysis of underreporting of tumor registration in a hospital in Shanghai. Article in Chinese. *Shanghai Journal of Preventive Medicine*. 2009;21(006):271-272. doi:10.3969/j.issn.1004-9231.2009.06.012

[33] National Health Commission of the People’s Republic of China. China Migrant Population Development Report 2018. China Population Publishing House; 2018.

[34] Lupo PJ, Schraw JM, Desrosiers TA, . Association between birth defects and cancer risk among children and adolescents in a population-based assessment of 10 million live births. JAMA Oncol. 2019;5(8):1150-1158. doi:10.1001/jamaoncol.2019.1215 31219523 PMC6587148

[35] Dai B, Yafei S, Hao X, Jiao L. Analysis of the results of a birth defects baseline survey in Inner Mongolian Autonomous Region. Article in Chinese. *Chinese Journal of Family Planning*. 2012;20(10):683-686. Accessed July 10, 2022. http://caod.oriprobe.com/articles/31090639/Analysis_of_the_results_of_a_birth_defects_baseline_survey_in_Inner_Mo.htm

[36] Hu R, Hu R, Zhang C, Hou X. Epidemiological survey of hypertension among Mongolians and Han Chinese in the pastoral areas of Inner Mongolia. Article in Chinese. *Practical Preventive Medicine*. 2005;12(5):2.

[37] He WQ, Gao X, Gao L, Ma Y, Sun D, Sun J. Contrasting trends of primary liver cancer mortality in Chinese Mongol and non-Mongol. Asian Pac J Cancer Prev. 2021;22(9):2757-2763. doi:10.31557/APJCP.2021.22.9.2757 34582643 PMC8850897

[38] Xin X, Bao BQ, Xi WD, . Survey and analysis of drinking among adults in Hulunbuir Mongolian region. Article in Chinese. *Modern Preventive Medicine*. 2013;40(4):697-700. Accessed July 10, 2022. http://caod.oriprobe.com/articles/38450250/hu_lun_bei_er_meng_gu_zu_di_qu_ju_min_yin_jiu_xing_wei_diao_cha_.htm

[39] Latino-Martel P, Chan DS, Druesne-Pecollo N, Barrandon E, Hercberg S, Norat T. Maternal alcohol consumption during pregnancy and risk of childhood leukemia: systematic review and meta-analysis. Cancer Epidemiol Biomarkers Prev. 2010;19(5):1238-1260. doi:10.1158/1055-9965.EPI-09-1110 20447918

[40] Deng ZY, Liu XJ, Sa RN, . Epidemiological investigation of allergic rhinitis in central cities and countrysides of Inner Mongolia Region. Article in Chinese. Zhonghua Er Bi Yan Hou Tou Jing Wai Ke Za Zhi. 2021;56(6):635-642. 34256489 10.3760/cma.j.cn115330-20200929-00778

[41] Wang XY, Zhuang Y, Ma TT, Zhang B, Wang XY. Prevalence of self-reported food allergy in six regions of Inner Mongolia, Northern China: a population-based survey. Med Sci Monit. 2018;24:1902-1911. doi:10.12659/MSM.908365 29605827 PMC5894567

[42] Wallace AD, Francis SS, Ma X, . Allergies and childhood acute lymphoblastic leukemia: a case-control study and meta-analysis. Cancer Epidemiol Biomarkers Prev. 2018;27(10):1142-1150. doi:10.1158/1055-9965.EPI-17-0584 30068517 PMC6628274

[43] Yeoh AE, Lu Y, Chan JY, . Genetic susceptibility to childhood acute lymphoblastic leukemia shows protection in Malay boys: results from the Malaysia-Singapore ALL Study Group. Leuk Res. 2010;34(3):276-283. doi:10.1016/j.leukres.2009.07.003 19651439

[44] Iqbal Z, Iqbal M, Akhter T. Frequency of BCR-ABL fusion oncogene in Pakistani childhood acute lymphoid leukemia (ALL) patients reflects ethnic differences in molecular genetics of ALL. J Pediatr Hematol Oncol. 2007;29(8):585. doi:10.1097/MPH.0b013e3180f61bcf 17762503

[45] Xu H, Yang W, Perez-Andreu V, . Novel susceptibility variants at 10p12.31-12.2 for childhood acute lymphoblastic leukemia in ethnically diverse populations. J Natl Cancer Inst. 2013;105(10):733-742. doi:10.1093/jnci/djt042 23512250 PMC3691938

[46] Gutierrez-Camino A, Martin-Guerrero I, García-Orad A. Genetic susceptibility in childhood acute lymphoblastic leukemia. Med Oncol. 2017;34(10):179. doi:10.1007/s12032-017-1038-7 28905228

[47] Yoo SK, Kim CU, Kim HL, . NARD: whole-genome reference panel of 1779 Northeast Asians improves imputation accuracy of rare and low-frequency variants. Genome Med. 2019;11(1):64-73. doi:10.1186/s13073-019-0677-z 31640730 PMC6805399

[48] Soliman R, Elhaddad A, Oke J, . Childhood cancer hospital resource utilization and costs in Egypt, 2013-2017; patterns, trends, and associated factors for 8886 patients from Children’s Cancer Hospital, Egypt. Pediatr Blood Cancer. 2021;68(11):e29347. doi:10.1002/pbc.29347 34520099

[49] de Oliveira C, Bremner KE, Liu N, . Costs for childhood and adolescent cancer, 90 days prediagnosis and 1 year postdiagnosis: a population-based study in Ontario, Canada. Value Health. 2017;20(3):345-356. doi:10.1016/j.jval.2016.10.010 28292479

[50] Zhou H, Wu Z, Wang H, . Analysis of the spectrum and characteristics of pediatric cancer based on hospital information systems in China. Cancer Manag Res. 2021;13:1205-1214. doi:10.2147/CMAR.S279427 33603466 PMC7884958

[51] Sui M, Zeng X, Tan WJ, . Catastrophic health expenditures of households living with pediatric leukemia in China. Cancer Med. 2020;9(18):6802-6812. doi:10.1002/cam4.3317 32697427 PMC7520357

[52] Chinese Red Cross Foundation. A report on the living conditions of children diagnosed with leukemia in rural China. Accessed July 10, 2022. https://pic.crcf.org.cn/attachment/20200413/19df9e4a481e411ba697b14904568047.pdf

[53] Friedrich P, Lam CG, Itriago E, Perez R, Ribeiro RC, Arora RS. Magnitude of Treatment Abandonment in Childhood Cancer. PLoS One. 2015;10(9):e0135230. doi:10.1371/journal.pone.0135230 26422208 PMC4589240

[54] Urenqiqig. Medical pluralism and human health—the medical anthropology investigation about the diversity of medical choices for contemporary Mongolians. Journal of the Central University for Nationalities. 2009;(1):22-27. Accessed July 10, 2022. http://caod.oriprobe.com/articles/46207889/Medical_Pluralism_and_Human_Health%E2%80%94%E2%80%94The_Medical_An.htm

[55] Yuan Y. Development of Mongolian population in Inner Mongolia. Article in Chinese. Inner Mongolia Statistics. 2015;(4):40-41. Accessed July 10, 2022. http://open.oriprobe.com/articles/46673088/nei_meng_gu_meng_gu_zu_ren_kou_de_fa_zhan_zhuang_k.htm

[56] Li M, Fan Y, McNeil EB, Chongsuvivatwong V. Traditional Mongolian, traditional Chinese, and Western medicine hospitals: system review and patient survey on expectations and perceptions of quality of healthcare in Inner Mongolia, China. Evid Based Complement Alternat Med. 2018;2018:2698461. doi:10.1155/2018/2698461 30108650 PMC6077555

[57] Yu M, Zhong J, Hu R, . The impact of catastrophic medical insurance in China: a five-year patient-level panel study. Lancet Reg Health West Pac. 2021;13:100174. doi:10.1016/j.lanwpc.2021.100174 34527979 PMC8358690

[58] Tian H, Xu R, Li F, . Identification of cancer patients using claims data from health insurance systems: a real-world comparative study. Chin J Cancer Res. 2019;31(4):699-706. doi:10.21147/j.issn.1000-9604.2019.04.13 31564812 PMC6736657

[59] Shi C, Liu M, Liu Z, . Using health insurance reimbursement data to identify incident cancer cases. J Clin Epidemiol. 2019;114:141-149. doi:10.1016/j.jclinepi.2019.06.009 31226412

