# Supplementary material for: Association of Ethnicity, Sex, and Age With Cancer Diagnoses and Health Care Utilization Among Children in Inner Mongolia, China
Source: JAMA Netw Open. 2022 Sep 12;5(9):e2231182. doi: 10.1001/jamanetworkopen.2022.31182 (PMC9468889; doi:10.1001/jamanetworkopen.2022.31182)
Supplement: Supplement. — eTable 1. Proportion of Garbage Codes in the Cause-of-Death-Reporting System in Inner Mongolia From 2014 to 2019 eTable 2. Incidence Rates of Childhood Cancer in Inner Mongolia During 2015-2019, Grouped by City and Ethnic Group, per Million Children eTable 3. Incidence Rates of Childhood Cancer in Inner Mongolia During 2015-2019, Grouped by GDP Per Capita Level/Grassland Area Share Level/Alcohol Consumption Rate Level/Smoking Rate Level, and Ethnic Group, per Million Children eTable 4. Five-Year Prevalence Rates of Childhood Cancer of 2018-2020 in Inner Mongolia, Grouped by Age, per Million Children eTable 5. Five-Year Prevalence Rates of Childhood Cancer of 2018-2020 in Inner Mongolia, Grouped by Sex and Ethnic Group, per Million Children eTable 6. Five-Year Prevalence Rates of Childhood Cancer of 2020 in Inner Mongolia, Grouped by Cancer Type, Sex, and Age, per Million Children eFigure 1. Five-Year Prevalence Rates of Childhood Cancer of 2018-2020 in Inner Mongolia, Grouped by City and Ethnic Group, per Million Children eTable 7. Five-Year Prevalence Rates of Childhood Cancer of 2018-2020 in Inner Mongolia, Grouped by GDP Per Capita Level/Grassland Area Share Level/Alcohol Consumption Rate Level/Smoking Rate Level, and Ethnic Group, per Million Children eTable 8. Survival Rates of Childhood Cancer of 2015-2019 in Inner Mongolia, Grouped by Year and Ethnic Group eTable 9. Medical Costs for Childhood Cancer Patients During the First Year and First 3 Years After Diagnosis in Inner Mongolia, 2015-2019, Grouped by City and Ethnic Group eTable 10. Medical Costs for Childhood Cancer Patients During the First Year and First 3 Years After Diagnosis in Inner Mongolia, 2015-2019, Grouped by GDP Per Capita Level/Grassland Area Share Level/Alcohol Consumption Rate Level/Smoking Rate Level, and Ethnic Group eFigure 2. Hospital Flow of Patients With Childhood Cancer During Cancer Treatment Course in Inner Mongolia, 2015-2019, Grouped by GDP Per Capita Level and Ethnic Group [file jamanetwopen-e2231182-s001.pdf]

Supplemental Online Content

Zhou HZW, Qiao LY, Zhang YJ, et al. Association of ethnicity, sex, and age with cancer diagnoses and health care utilization among children in Inner Mongolia, China. *JAMA Netw Open*. 2022;5(9):e2231182. doi:10.1001/jamanetworkopen.2022.31182

eTable 1. Proportion of Garbage Codes in the Cause-of-Death-Reporting System in Inner Mongolia From 2014 to 2019
eTable 2. Incidence Rates of Childhood Cancer in Inner Mongolia during 2015-2019, Grouped by City and Ethnic Group, per Million Children
eTable 3. Incidence Rates of Childhood Cancer in Inner Mongolia During 2015-2019, Grouped by GDP Per Capita Level/Grassland Area Share Level/Alcohol Consumption Rate Level/Smoking Rate Level, and Ethnic Group, per Million Children
eTable 4. Five-Year Prevalence Rates of Childhood Cancer of 2018-2020 in Inner Mongolia, Grouped by Age, per Million Children
eTable 5. Five-Year Prevalence Rates of Childhood Cancer of 2018-2020 in Inner Mongolia, Grouped by Sex and Ethnic Group, per Million Children
eTable 6. Five-Year Prevalence Rates of Childhood Cancer of 2020 in Inner Mongolia, Grouped by Cancer Type, Sex, and age, per Million Children
eFigure 1. Five-Year Prevalence Rates of Childhood Cancer of 2018-2020 in Inner Mongolia, Grouped by City and Ethnic Group, per Million Children
eTable 7. Five-Year Prevalence Rates of Childhood Cancer of 2018-2020 in Inner Mongolia, Grouped by Gdp Per Capita Level/Grassland Area Share Level/Alcohol Consumption Rate Level/Smoking Rate Level, and Ethnic Group, per Million Children
eTable 8. Survival Rates of Childhood Cancer of 2015-2019 in Inner Mongolia, Grouped by Year and Ethnic Group
eTable 9. Medical Costs for Childhood Cancer Patients During the First Year and First 3 Years After Diagnosis in Inner Mongolia, 2015-2019, Grouped by City and Ethnic Group
eTable 10. Medical Costs for Childhood Cancer Patients During the First Year and First 3 Years After Diagnosis in Inner Mongolia, 2015-2019, Grouped by GDP Per Capita Level/Grassland Area Share Level/Alcohol Consumption Rate Level/Smoking Rate Level, and Ethnic Group
eFigure 2. Hospital Flow of Patients With Childhood Cancer During Cancer Treatment Course in Inner Mongolia, 2015-2019, Grouped by GDP Per Capita Level and Ethnic Group

This supplemental material has been provided by the authors to give readers additional information about their work.

© 2022 Zhou HZW et al. *JAMA Network Open.*

eTable 1. Proportion of Garbage Codes in the Cause-of-Death-Reporting System in Inner Mongolia From 2014 to 2019

**Accuracy Indexes**	**2014**	**2015**	**2016**	**2017**	**2018**	**2019**
Proportion of unknown cause of mortality (%)	1.19	0.84	0.55	0.57	0.55	0.67
Proportion of undetermined intent of injury (%)	0.10	0.11	0.11	0.12	0.15	0.13
Proportion of unspecified heart disease (%)	1.73	1.74	1.58	1.13	1.03	1.03
Proportion of neoplasms with unspecified site (%)	0.09	0.03	0.06	0.06	0.06	0.08
Proportion of respiratory failure or liver failure (%)	0.42	0.27	0.24	0.39	0.52	0.28
Total (%)	3.53	2.99	2.54	2.27	2.31	2.19

© 2022 Zhou HZW et al. *JAMA Network Open.*

eTable 2. Incidence Rates of Childhood Cancer in Inner Mongolia during 2015-2019, Grouped by City and Ethnic Group, per Million Children[a]

**City**	Han			Mongolian		
	**Incidence**	**95% CI (Lower)**	**95% CI (Upper)**	**Incidence**	**95% CI (Lower)**	**95% CI (Upper)**
Alxa League	212.67	108.47	316.87	150.14	0.00	320.03
Bayannur	128.49	107.05	149.92	60.18	0.00	143.57
Baotou	123.75	104.07	143.42	99.95	0.00	295.84
Chifeng	174.28	155.94	192.61	242.37	143.33	341.41
Ordos	64.46	50.83	78.08	92.83	42.37	143.29
Hohhot	95.21	77.73	112.69	65.00	19.96	110.03
Hulun Buir	101.02	81.79	120.25	66.71	20.48	112.93
Tongliao	234.59	192.08	277.09	202.04	172.11	231.97
Wuhai	132.00	86.27	177.74	122.03	0.00	291.15
Ulanqab	131.79	108.60	154.99	314.56	0.00	670.47
Xilingol League	134.49	101.02	167.97	65.23	20.03	110.43
Hinggan League	109.93	84.36	135.49	108.88	65.32	152.44

[a]All results were crude incidence rates.

CI, confidence interval.

© 2022 Zhou HZW et al. *JAMA Network Open.*

eTable 3. Incidence Rates of Childhood Cancer in Inner Mongolia During 2015-2019, Grouped by GDP Per Capita Level/Grassland Area Share Level/Alcohol Consumption Rate Level/Smoking Rate Level, and Ethnic Group, per Million Children

**Subgroups**	**Incidence (95% CI), per million children**	
	Han	Mongolian
**GDP per capita level**[a]		
Low (< $9,265)	145.65 (136.15-155.14)	174.82 (152.47-197.18)
High (> $9,265)	101.79 (92.51-111.08)	80.99 (54.16-107.82)
**Grassland area share level**[b]		
Low (< 64.55%)	139.01 (129.82-148.20)	168.06 (146.84-189.27)
High (> 64.55%)	110.07 (100.29-119.86)	84.72 (53.89-115.56)
**Alcohol consumption rate level**[c]		
Low (< 30.85%)	104.43 (95.61-113.25)	84.61 (53.82-115.4)
High (> 30.85%)	148.02 (137.93-158.11)	168.11 (146.89-189.33)
**Smoking rate level**[d]		
Low (< 25.47%)	111.54 (101.38-121.7)	94.24 (67.58-120.9)
High (> 25.47%)	136.93 (127.99-145.87)	175.22 (152.17-198.27)

[a]The median GDP per capita of cities in Inner Mongolia was $9,265 in 2019. High-level city group (GDP per capita>$9,265) includes Alxa League, Wuhai, Baotou, Hohhot, Ordos and Xilin Gol League, low-level city group (GDP per capita<$9,265) includes Bayannur, Hulun Buir, Tongliao, Chifeng, Ulanqab, and Hinggan League.

[b]The median percentage of grassland area share of cities in Inner Mongolia is 64.55%. High-level city group (grassland area share >64.55%) includes Ordos, Alxa league, Wuhai, Baotou, Bayannur, and Xilin Gol league, low-level city group (grassland area share <64.55%) includes Hulun Buir, Hohhot, Tongliao, Chifeng, Ulanqab, and Hinggan league.

[c]The median city-level adult alcohol consumption rate in Inner Mongolia was 30.85% in 2018. High-level city group (alcohol consumption rate >30.85%) includes Chifeng, Tongliao, Xilin Gol league, Hulun Buir, Hinggan league and Ulanqab, low-level city group (alcohol consumption rate <30.85%) includes Ordos, Baotou, Bayannur, Hohhot, Alxa league and Wuhai.

[d]The median city-level adult smoking rate in Inner Mongolia was 25.47% in 2018. High-level city group (smoking rate >25.47%) includes Ordos, Baotou, Bayannur, Chifeng, Tongliao and Xilin Gol league, low-level city group (smoking rate <25.47%) includes Hohhot, Alxa league, Wuhai, Hulun Buir, Hinggan league and Ulanqab.

CI, confidence interval; GDP, gross domestic product.

© 2022 Zhou HZW et al. *JAMA Network Open.*

eTable 4. Five-Year Prevalence Rates of Childhood Cancer of 2018-2020 in Inner Mongolia, Grouped by Age, per Million Children

Year		Overall[a]	Age, y		
			0–4	5–9	10–14
2018	**Crude Rate (95% CI)**	259.59 (240.87-278.32)	307.98 (269.34-346.62)	270.41 (238.57-302.25)	211.34 (183.23-239.46)
	**WSR (95% CI)**	263.75 (244.87-282.63)	/	/	/
	**Adjusted Rate (95% CI)** [b]	253.97 (235.44-272.49)	299.15 (261.07-337.23)	266.51 (234.90-298.11)	205.50 (177.77-233.22)
2019	**Crude Rate (95% CI)**	370.87 (348.72-393.01)	440.54 (394.72-486.35)	410.55 (371.71-449.4)	278.19 (246.34-310.04)
	**WSR (95% CI)**	377.18 (354.85-399.51)	/	/	/
	**Adjusted Rate (95% CI)** [b]	360.54 (338.70-382.37)	426.88 (381.78-471.99)	404.81 (366.24-443.38)	273.44 (241.87-305.02)
2020	**Crude Rate (95% CI)**	425.49 (402.13-448.84)	473.45 (426.35-520.55)	470.77 (430.51-511.03)	340.91 (305.80-376.02)
	**WSR (95% CI)**	428.97 (405.52-452.42)	/	/	/
	**Adjusted Rate (95% CI)** [b]	410.80 (387.86-433.75)	457.59 (411.29-503.89)	461.80 (421.92-501.68)	333.38 (298.65-368.10)

[a]The overall consisted of incident cases from Han, Mongolian, and Other ethnic minorities. Other minorities were not included in the ethnicity-specific analysis to obtain a relatively reliable estimation of the rate because of the small sample size of patients.

[b]The number of patients diagnosed that died during 2015-2019 was recalculated based on underreporting rate from the two cause-of-death reporting system completeness surveys (7.85% in 2015-2017 and 5.65% in 2018-2020). Adjustments were made to the crude rate.

CI, confidence interval; WSR: Age-standardized rate by world standard population (WHO 2000-2025).

.

© 2022 Zhou HZW et al. *JAMA Network Open.*

eTable 5. Five-Year Prevalence Rates of Childhood Cancer of 2018-2020 in Inner Mongolia, Grouped by Sex and Ethnic Group, per Million Children

Year		Sex		Ethnic group	
		Male	Female	Han	Mongolian
2018	**Crude Rate (95% CI)**	286.30 (259.15-313.45)	230.12 (204.55-255.69)	253.17 (232.91-273.42)	285.27 (233.14-337.40)
	**WSR (95% CI)**	289.17 (261.88-316.46)	235.76 (209.88-261.64)	258.26 (237.80-278.72)	285.81 (233.63-337.99)
	**Adjusted Rate (95% CI)** [a]	278.26 (251.49-305.02)	222.72 (197.56-247.88)	246.42 (226.43-326.57)	275.35 (224.13-326.57)
2019	**Crude Rate (95% CI)**	401.57 (369.73-433.4)	337.81 (307.14-368.48)	356.99 (333.39-380.59)	454.21 (386.54-521.88)
	**WSR (95% CI)**	405.13 (373.19-437.07)	346.46 (315.40-377.52)	365.07 (341.21-388.93)	453.85 (386.21-521.49)
	**Adjusted Rate (95% CI)** [a]	392.37 (360.90-423.83)	327.66 (297.46-357.86)	348.87 (325.54-372.19)	441.08 (374.40-507.77)
2020	**Crude Rate (95% CI)**	452.90 (419.64-486.16)	395.23 (362.59-427.87)	404.34 (379.77-428.91)	568.49 (491.62-645.36)
	**WSR (95% CI)**	454.21 (420.9-487.52)	401.26 (368.37-434.15)	408.65 (383.95-433.35)	569.29 (492.37-646.21)
	**Adjusted Rate (95% CI)** [a]	440.18 (407.39-472.97)	382.59 (350.48-414.71)	395.40 (371.10-419.69)	549.54 (473.96-625.12）

[a]The number of patients diagnosed that died during 2015-2019 was recalculated based on underreporting rate from the two cause-of-death reporting system completeness surveys (7.85% in 2015-2017 and 5.65% in 2018-2020). Adjustments were made to the crude rate.

CI, confidence interval; WSR: Age-standardized rate by world standard population (WHO 2000-2025).

© 2022 Zhou HZW et al. *JAMA Network Open.*

eTable 6. Five-Year Prevalence Rates of Childhood Cancer of 2020 in Inner Mongolia, Grouped by Cancer Type, Sex, and age, per Million Children

Sex	Diagnosis subtypes	Overall		0–4 years		5–9 years		10–14 years	
		No.	WSR	No.	ASR	No.	ASR	No.	ASR
Male	Overall	712	454.21 (420.9-487.52)	210	481.93 (416.76-547.09)	308	528.99 (469.93-588.05)	194	350.12 (300.86-399.38)
	Leukemia	272	167.68 (147.44-187.92)	53	121.63 (88.89-154.37)	154	264.49 (222.72-306.26)	65	117.31 (88.79-145.82)
	Lymphomas	50	31.02 (22.31-39.73)	10	22.95 (8.73-37.17)	22	37.78 (22.00-53.57)	18	32.49 (17.48-47.49)
	CNS tumors	77	48.67 (37.76-59.58)	20	45.90 (25.78-66.01)	31	53.24 (34.50-71.98)	26	46.92 (28.89-64.96)
	Sympathetic nervous system tumors	36	24.46 (16.73-32.19)	18	41.31 (22.23-60.39)	10	17.17 (6.53-27.82)	8	14.44 (4.43-24.44)
	Retinoblastoma	24	17.02 (10.57-23.47)	16	36.72 (18.73-54.71)	7	12.02 (3.12-20.93)	1	1.80 (0.00-5.34)
	Renal tumors	15	10.83 (5.69-15.97)	11	25.24 (10.33-40.16)	4	6.87 (0.14-13.6)	0	0
	Hepatic tumors	14	9.66 (4.80-14.52)	8	18.36 (5.64-31.08)	5	8.59 (1.06-16.11)	1	1.80 (0.00-5.34)
	Bone tumors	22	13.13 (7.47-18.79)	1	2.29 (0.00-6.79)	5	8.59 (1.06-16.11)	16	28.88 (14.73-43.02)
	Soft tissue sarcomas	18	11.19 (5.96-16.42)	4	9.18 (0.18-18.18)	10	17.17 (6.53-27.82)	4	7.22 (0.14-14.29)
	Germ cell and gonadal tumors	13	8.10 (3.65-12.55)	3	6.88 (0.00-14.68)	7	12.02 (3.12-20.93)	3	5.41 (0.00-11.54)
	Epithelial tumors and melanoma	85	55.96 (44.27-67.65)	33	75.73 (49.89-101.57)	25	42.94 (26.11-59.77)	27	48.73 (30.35-67.11)
	Other and Unspecified	86	56.48 (44.73-68.23)	33	75.73 (49.89-101.57)	28	48.09 (30.28-65.90)	25	45.12 (27.43-62.80)

© 2022 Zhou HZW et al. *JAMA Network Open.*

Sex	Diagnosis subtypes	Overall		0–4 years		5–9 years		10–14 years	
		No.	WSR	No.	ASR	No.	ASR	No.	ASR
Female	Overall	563	401.26 (368.37-434.15)	178	463.83 (395.71-531.96)	217	407.16 (353-461.32)	168	330.86 (280.84-380.89)
	Leukemia	191	131.18 (112.37-149.99)	41	106.84 (74.14-139.54)	90	168.87 (133.98-203.75)	60	118.16 (88.27-148.06)
	Lymphomas	32	23.28 (15.36-31.20)	12	31.27 (13.58-48.96)	11	20.64 (8.44-32.84)	9	17.72 (6.14-29.30)
	CNS tumors	56	37.92 (27.81-48.03)	10	26.06 (9.91-42.21)	29	54.41 (34.61-74.22)	17	33.48 (17.56-49.40)
	Sympathetic nervous system tumors	39	28.65 (19.86-37.44)	16	41.69 (21.26-62.12)	16	30.02 (15.31-44.73)	7	13.79 (3.57-24.00)
	Retinoblastoma	23	18.25 (11.23-25.27)	15	39.09 (19.31-58.87)	7	13.13 (3.4-22.86)	1	1.97 (0.00-5.83)
	Renal tumors	12	9.87 (4.71-15.03)	9	23.45 (8.13-38.77)	1	1.88 (0.00-5.55)	2	3.94 (0.00-9.4)
	Hepatic tumors	8	6.57 (2.36-10.78)	6	15.63 (3.12-28.15)	1	1.88 (0.00-5.55)	1	1.97 (0.00-5.83)
	Bone tumors	20	13.82 (7.72-19.92)	4	10.42 (0.21-20.64)	3	5.63 (0.00-12.00)	13	25.6 (11.68-39.52)
	Soft tissue sarcomas	17	12.04 (6.34-17.74)	5	13.03 (1.61-24.45)	6	11.26 (2.25-20.27)	6	11.82 (2.36-21.27)
	Germ cell and gonadal tumors	39	27.67 (19.03-36.31)	11	28.66 (11.72-45.6)	7	13.13 (3.40-22.86)	21	41.36 (23.67-59.05)
	Epithelial tumors and melanoma	60	42.46 (31.76-53.16)	18	46.90 (25.24-68.57)	26	48.78 (30.03-67.54)	16	31.51 (16.07-46.95)
	Other and Unspecified	66	49.55 (37.99-61.11)	31	80.78 (52.34-109.21)	20	37.53 (21.08-53.97)	15	29.54 (14.59-44.49)
Overall		1275	428.97 (405.52-452.42)	388	473.45 (426.35-520.55)	525	470.77 (430.51-511.03)	362	340.91 (305.80-376.02)

WSR: World Standard population age-standardized Rates (WHO 2000-2025); ASR: Age-Specific Rates; CNS, central nervous system.

© 2022 Zhou HZW et al. *JAMA Network Open.*

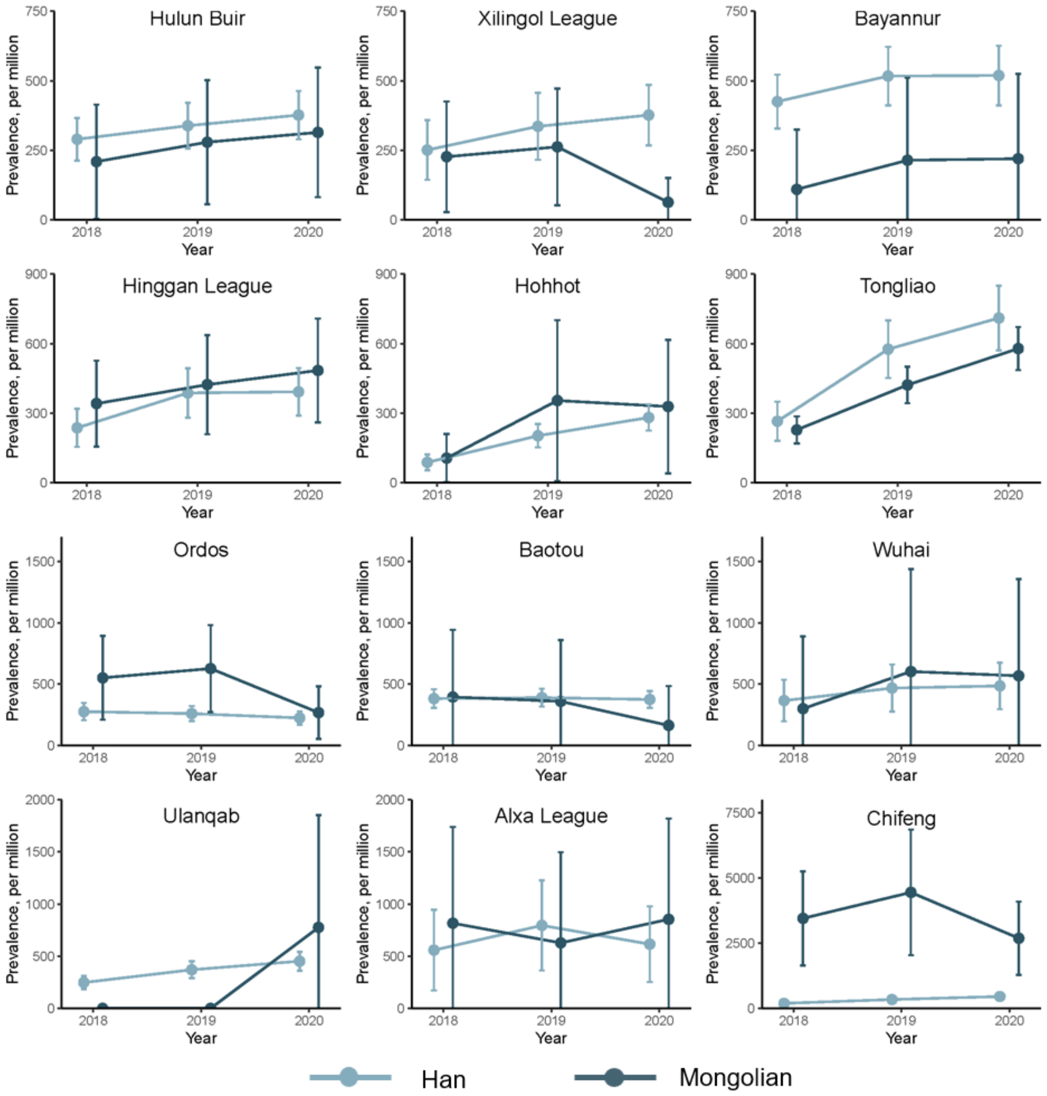

eFigure 1. Five-Year Prevalence Rates of Childhood Cancer of 2018-2020 in Inner Mongolia, Grouped by City and Ethnic Group, per Million Children[a]

[a]All results are crude 5-year prevalence rates, and the error lines in each bar chart show 95% confidence intervals.

© 2022 Zhou HZW et al. *JAMA Network Open.*

eTable 7. Five-Year Prevalence Rates of Childhood Cancer of 2018-2020 in Inner Mongolia, Grouped by Gdp Per Capita Level/Grassland Area Share Level/Alcohol Consumption Rate Level/Smoking Rate Level, and Ethnic Group, per Million Children[a]

Year	Subgroups	5-year prevalence rate (95% CI)	
		Han	Mongolian
2018	**GDP per capita level[b]**		
	Low (< $9,265)	253.13 (227.41-278.85)	282.44 (224.10-340.78)
	High (> $9,265)	253.24 (220.37-286.10)	295.96 (179.96-411.96)
	**Grassland area share level[c]**		
	Low (< 64.55%)	204.28 (182.01-226.56)	262.56 (209.21-315.92)
	High (> 64.55%)	351.16 (309.81-392.50)	449.70 (261.82-637.57)
	**Alcohol consumption rate level[d]**		
	Low (< 30.85%)	283.62 (250.46-316.78)	328.10 (187.79-468.41)
	High (> 30.85%)	231.29 (205.91-256.67)	277.19 (221.16-333.22)
	**Smoking rate level[e]**		
	Low (< 25.47%)	215.79 (185.58-246.00)	244.20 (148.48-339.92)
	High (> 25.47%)	276.40 (249.45-303.35)	299.25 (237.43-361.07)
2019	**GDP per capita level[b]**		
	Low (< $9,265)	393.35 (361.15-425.55)	450.67 (377.33-524.00)
	High (> $9,265)	304.31 (270.22-338.41)	473.48 (298.15-648.82)
	**Grassland area share level[c]**		
	Low (< 64.55%)	342.58 (313.77-371.39)	450.67 (373.34-517.28)
	High (> 64.55%)	383.03 (342.08-423.98)	512.08 (315.30-708.87)
	**Alcohol consumption rate level[d]**		
	Low (< 30.85%)	336.30 (301.95-370.65)	635.83 (381.53-890.13)
	High (> 30.85%)	373.54 (341.16-405.92)	434.23 (364.52-503.94)
	**Smoking rate level[e]**		
	Low (< 25.47%)	322.78 (286.01-359.55)	385.62 (245.30-525.94)
	High (> 25.47%)	377.29 (346.67-407.91)	471.08 (394.15-548.01)
2020	**GDP per capita level[b]**		
	Low (< $9,265)	470.75 (435.50-505.99)	588.59 (505.14-672.04)
	High (> $9,265)	317.83 (284.77-350.89)	423.21 (232.95-613.47)
	**Grassland area share level[c]**		
	Low (< 64.55%)	426.37 (394.54-458.20)	577.00 (495.82-685.17)
	High (> 64.55%)	367.11 (328.71-405.51)	482.28 (246.02-718.53)
	**Alcohol consumption rate level[d]**		
	Low (< 30.85%)	341.53 (308.02-375.04)	420.14 (231.26-609.02)
	High (> 30.85%)	456.61 (421.27-491.95)	589.18 (505.65-672.71)
	**Smoking rate level[e]**		
	Low (< 25.47%)	372.70 (333.88-411.52)	450.25 (305.2-595.30)
	High (> 25.47%)	422.87 (391.23-454.51)	602.32 (512.59-692.05)

© 2022 Zhou HZW et al. *JAMA Network Open.*

[a]All results were crude 5-year prevalence rates.

[b]The median GDP per capita of cities in Inner Mongolia was $9,265 in 2019. High-level city group (GDP per capita>$9,265) includes Alxa League, Wuhai, Baotou, Hohhot, Ordos and Xilin Gol League, low-level city group (GDP per capita<$9,265) includes Bayannur, Hulun Buir, Tongliao, Chifeng, Ulanqab, and Hinggan League.

[c]The median percentage of grassland area share of cities in Inner Mongolia is 64.55%. High-level city group (grassland area share >64.55%) includes Ordos, Alxa league, Wuhai, Baotou, Bayannur, and Xilin Gol league, low-level city group (grassland area share <64.55%) includes Hulun Buir, Hohhot, Tongliao, Chifeng, Ulanqab, and Hinggan league.

[d]The median city-level adult alcohol consumption rate in Inner Mongolia was 30.85% in 2018. High-level city group (alcohol consumption rate >30.85%) includes Chifeng, Tongliao, Xilin Gol league, Hulun Buir, Hinggan league and Ulanqab, low-level city group (alcohol consumption rate <30.85%) includes Ordos, Baotou, Bayannur, Hohhot, Alxa league and Wuhai.

[e]The median city-level adult smoking rate in Inner Mongolia was 25.47% in 2018. High-level city group (smoking rate >25.47%) includes Ordos, Baotou, Bayannur, Chifeng, Tongliao and Xilin Gol league, low-level city group (smoking rate 25.47%) includes Hohhot, Alxa league, Wuhai, Hulun Buir, Hinggan league and Ulanqab.

CI, confidence interval; GDP, gross domestic product.

© 2022 Zhou HZW et al. *JAMA Network Open.*

eTable 8. Survival Rates of Childhood Cancer of 2015-2019 in Inner Mongolia, Grouped by Year and Ethnic Group

	Year	Unadjusted survival rate (%)			Adjusted survival rate (%)[a]		
		Overall[b]	Han	Mongolian	Overall[b]	Han	Mongolian
5-year	2015	70.59 (120/170)	73.19 (101/138)	61 (14/23)	67.65 (115/170)	71.01 (98/138)	61 (14/23)
3-year	2015	72.94 (124/170)	74.64 (103/138)	65 (15/23)	70.00 (119/170)	72.46 (100/138)	65 (15/23)
	2016	63.90 (177/277)	63.35 (140/221)	68 (27/40)	61.10 (170/277)	60.18 (133/221)	65 (26/40)
	2017	64.72 (310/479)	63.36 (249/393)	73 (59/81)	62.00 (297/479)	60.56 (238/393)	70 (57/81)
	Combined (95% CI)[c]	66.77 (61.62-71.92)	66.76 (59.75-73.78)	70.32 (62.88-77.76)	63.96 (59.12-68.80)	64.05 (56.53-71.58)	68.17 (60.57-75.76)
1-year	2015	80.59 (137/170)	81.16 (112/138)	78 (18/23)	78.82 (134/170)	79.71 (110/138)	78 (18/23)
	2016	72.56 (201/277)	72.40 (160/221)	78 (31/40)	70.40 (195/277)	70.14 (155/221)	75 (30/40)
	2017	70.15 (336/479)	69.47 (273/393)	74 (60/81)	67.64 (324/479)	66.92 (263/393)	72 (58/81)
	2018	75.25 (307/408)	74.40 (250/336)	79 (53/67)	73.77 (301/408)	72.92 (245/336)	78 (52/67)
	2019	64.62 (221/342)	63.18 (175/277)	69 (41/59)	62.57 (214/342)	61.01 (169/277)	68 (40/59)
	Combined (95% CI)[c]	72.52 (67.51-77.53)	72.00 (66.43-77.57)	75.51 (70.40-80.63)	70.52 (65.30-75.73)	70.01 (64.12-75.89)	73.68 (68.45-78.91)

[a]The number of patients diagnosed that died during 2015-2019 was recalculated based on underreporting rate from the two cause-of-death reporting system completeness surveys (7.85% in 2015-2017 and 5.65% in 2018-2020).

[b]The overall consisted of incident cases from Han, Mongolian, and Other ethnic minorities. Other minorities were not included in the ethnicity-specific analysis to obtain a relatively reliable estimation of the rate because of the small sample size of patients.

[c]Survival rates were meta-combined using a random-effects model.

CI, confidence interval.

© 2022 Zhou HZW et al. *JAMA Network Open.*

eTable 9. Medical Costs for Childhood Cancer Patients During the First Year and First 3 Years After Diagnosis in Inner Mongolia, 2015-2019, Grouped by City and Ethnic Group[a]

City	Cost, median (IQR), $a			
	Han		Mongolian	
	1 y post-diagnosis	3 y post-diagnosis	1 y post-diagnosis	3 y post-diagnosis
Alxa League	3,084 (1,366-8,869)	4,796 (1,457-20,922)	19,816 (19,350-24,026)	24,651 (31,767-39,976)
Bayannur	1,880 (1,007-12,423)	2,894 (1,060-17,194)	10,894 (8,234-13,553)	48,926 (21,439-56,413)
Baotou[b]	6,434 (1,951-19,097)	13,097 (1,378-28,759)	47,770 (47,770-47,770)	48,126 (18,126-48,126)
Chifeng	5,607 (1,142-17,065)	6,624 (3,218-24,509)	1,485 (805-16,669)	2,252 (2,086-25,766)
Ordos	3,765 (2,538-16,406)	5,323 (8,586-23,936)	2,586 (1,029-7,614)	5,173 (1,029-7,984)
Hohhot	3,100 (1,075-9,499)	3,825 (1,084-12,008)	1,821 (1,353-2,388)	1,821 (8,353-6,227)
Hulun Buir	2,920 (921-9,282)	3,514 (1,065-13,986)	1,160 (893-5,712)	1,160 (893-5,989)
Tongliao	1,854 (746-9,538)	2,457 (160-16,424)	1,588 (789-7,670)	1,861 (144-10,563)
Wuhai	7,234 (2,517-15,948)	11,378 (3,517-32,466)	20,899 (17,363-24,434)	22,092 (29,152-25,031)
Ulanqab	5,081 (1,218-14,762)	6,962 (1,308-20,769)	2,280 (1,433-24,409)	2,280 (3,433-24,409)
Xilingol League	4,165 (1,448-13,098)	6,505 (6,038-15,366)	2,506 (2,024-6,104)	7,707 (8,568-12,044)
Hinggan League	10,042 (2,094-18,972)	15,052 (7,614-29,373)	4,988 (1,529-20,638)	7,098 (2,675-22,964)

[a]Costs are discounted based on the Consumer Price Index; the RMB-to-USD exchange rate is based on the July 1, 2019, exchange rate (1.00 USD = 6.87 RMB).

[b]Due to small population size of Baotou, only one Mongolian case occurred during 2015-2019, resulting in its median costs were equal to the quartile.

IQR, interquartile range.

© 2022 Zhou HZW et al. *JAMA Network Open.*

eTable 10. Medical Costs for Childhood Cancer Patients During the First Year and First 3 Years After Diagnosis in Inner Mongolia, 2015-2019, Grouped by GDP Per Capita Level/Grassland Area Share Level/Alcohol Consumption Rate Level/Smoking Rate Level, and Ethnic Group[a]

Subgroups	Cost, median (IQR), $a			
	Han		Han	
	1 y post-diagnosis	1 y post-diagnosis	1 y post-diagnosis	1 y post-diagnosis
**GDP per capita level**[b]				
Low (< $9,265)	3,857 (1,063-15,432)	5,004 (1,155-22,155)	1,786 (838-9,937)	2,362 (935-13,115)
High (> $9,265)	4,424 (1,667-15,422)	6,755 (1,833-23,217)	2,630 (1,520-14,268)	5,173 (1,821-17,548)
**Grassland area share level**[c]				
Low (< 64.55%)	3,879 (1,073-15,360)	5,194 (1,149-21,750)	1,712 (842-9,476)	2,252 (940-13,058)
High (> 64.55%)	4,077 (1,408-15,518)	6,435 (1,616-23,929)	5,173 (2,383-15,373)	10,451 (2,538-24,651)
**Alcohol consumption rate level**[d]				
Low (< 30.85%)	3,797 (1,288-14,485)	5,316 (1,476-22,459)	5,173 (1,629-15,803)	7,614 (1,629-24,651)
High (> 30.85%)	4,378 (1,083-15,556)	5,541 (1,207-22,475)	1,792 (842-9,530)	2,557 (940-13,058)
**Smoking rate level**[e]				
Low (< 25.47%)	3,864 (1,246-13,430)	5,310 (1,371-20,532)	3,871 (1,315-19,116)	4,988 (1,382-22,369)
High (> 25.47%)	4,077 (1,153-16,090)	5,562 (1,273-23,454)	1,812 (836-9,011)	2,516 (924-13,098)

[a]Costs are discounted based on the Consumer Price Index; the RMB-to-USD exchange rate is based on the July 1, 2019, exchange rate (1.00 USD = 6.87 RMB)

[b]The median GDP per capita of cities in Inner Mongolia was $9,265 in 2019. High-level city group (GDP per capita>$9,265) includes Alxa League, Wuhai, Baotou, Hohhot, Ordos and Xilin Gol League, low-level city group (GDP per capita<$9,265) includes Bayannur, Hulun Buir, Tongliao, Chifeng, Ulanqab, and Hinggan League.

[c]The median percentage of grassland area share of cities in Inner Mongolia is 64.55%. High-level city group (grassland area share >64.55%) includes Ordos, Alxa league, Wuhai, Baotou, Bayannur, and Xilin Gol league, low-level city group (grassland area share <64.55%) includes Hulun Buir, Hohhot, Tongliao, Chifeng, Ulanqab, and Hinggan league.

[d]The median city-level adult alcohol consumption rate in Inner Mongolia was 30.85% in 201. High-level city group (alcohol consumption rate >30.85%) includes Chifeng, Tongliao, Xilin Gol league, Hulun Buir, Hinggan league and Ulanqab, low-level city group (alcohol consumption rate <30.85%) includes Ordos, Baotou, Bayannur, Hohhot, Alxa league and Wuhai.

[e]The median city-level adult smoking rate in Inner Mongolia was 25.47% in 2018. High-level city group (smoking rate >25.47%) includes Ordos, Baotou, Bayannur, Chifeng, Tongliao and Xilin Gol league, low-level city group (smoking rate <25.47%) includes Hohhot, Alxa league, Wuhai, Hulun Buir, Hinggan league and Ulanqab.

IQR, interquartile range.

© 2022 Zhou HZW et al. *JAMA Network Open.*

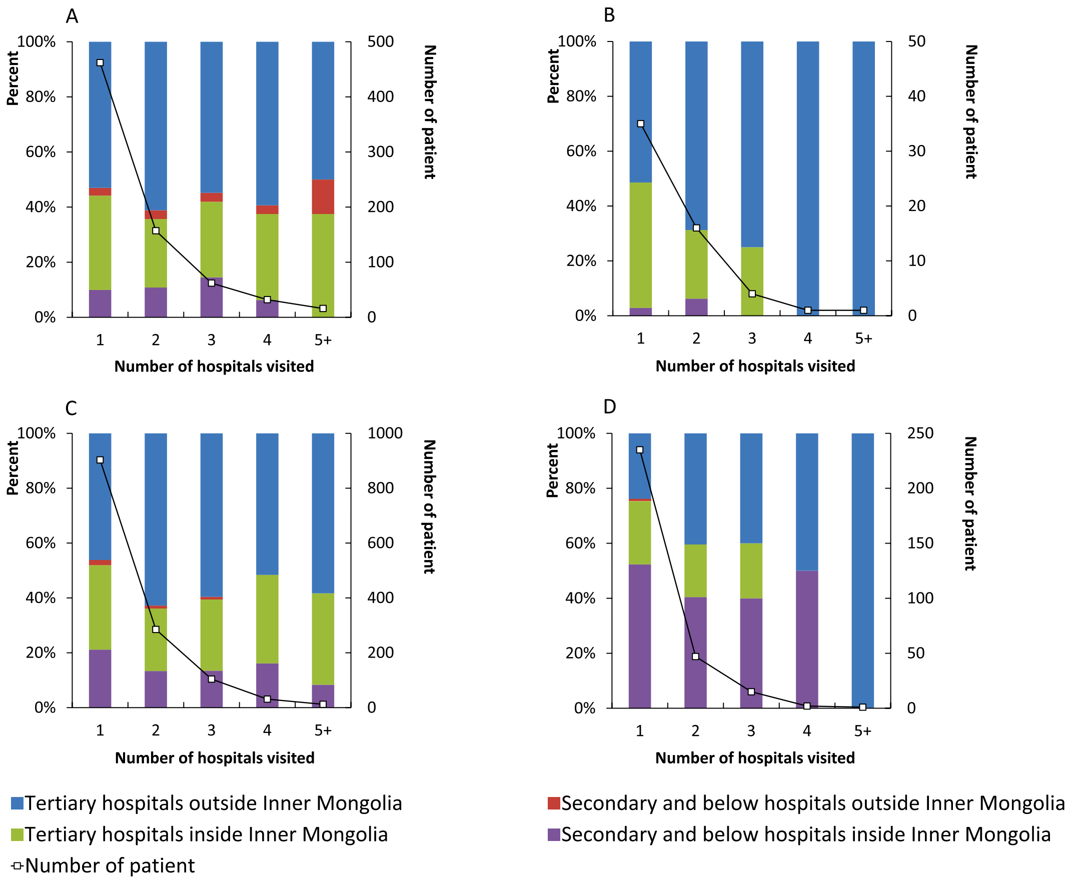

eFigure 2. Hospital Flow of Patients With Childhood Cancer During Cancer Treatment Course in Inner Mongolia, 2015-2019, Grouped by GDP Per Capita Level and Ethnic Group[a]

The solid black line shows the number of patients who visited the corresponding number of hospitals during the entire course of cancer treatment.

[a]The median GDP per capita of cities in Inner Mongolia was $9,265 in 2019. High-level city group (GDP per capita>$9,265) includes Alxa League, Wuhai, Baotou, Hohhot, Ordos and Xilin Gol League, low-level city group (GDP per capita<$9,265) includes Bayannur, Hulun Buir, Tongliao, Chifeng, Ulanqab, and Hinggan League.

(A) Han patients in high-level city group; (B) Mongolian patients in high-level city group; (C) Han patients in low-level city group; (D) Mongolian patients in low-level city group.

GDP, gross domestic product.

© 2022 Zhou HZW et al. *JAMA Network Open.*
